# Recent Advances in the Discovery and Development of Marine Microbial Natural Products

**DOI:** 10.3390/md11030700

**Published:** 2013-03-08

**Authors:** Zhi-Qiang Xiong, Jian-Feng Wang, Yu-You Hao, Yong Wang

**Affiliations:** 1 Key Laboratory of Synthetic Biology, Institute of Plant Physiology and Ecology, Shanghai Institutes for Biological Sciences, Chinese Academy of Sciences, Shanghai 200032, China; E-Mails: zhqxiong@sibs.ac.cn (Z.-Q.X.); wjfecust@yahoo.com.cn (J.-F.W.); yyhao@sibs.ac.cn (Y.-Y.H.); 2 State Key Laboratory of Bioreactor Engineering, East China University of Science and Technology, Shanghai 200237, China

**Keywords:** marine microbial natural products, metagenomics, screening, genome sequencing, combinatorial biosynthesis, heterologous biosynthesis

## Abstract

Marine microbial natural products (MMNPs) have attracted increasing attention from microbiologists, taxonomists, ecologists, agronomists, chemists and evolutionary biologists during the last few decades. Numerous studies have indicated that diverse marine microbes appear to have the capacity to produce an impressive array of MMNPs exhibiting a wide variety of biological activities such as antimicrobial, anti-tumor, anti-inflammatory and anti-cardiovascular agents. Marine microorganisms represent an underexplored reservoir for the discovery of MMNPs with unique scaffolds and for exploitation in the pharmaceutical and agricultural industries. This review focuses on MMNPs discovery and development over the past decades, including innovative isolation and culture methods, strategies for discovering novel MMNPs via routine screenings, metagenomics, genomics, combinatorial biosynthesis, and synthetic biology. The potential problems and future directions for exploring MMNPs are also discussed.

## 1. Introduction

There is a perpetual need for new chemotherapeutants, especially novel antibiotics, to combat new diseases and drug-resistant pathogens that are becoming a significant threat to public health [1]. The discovery and development of new drugs from natural products (NPs) has played a significant role over the last few decades. Over 28% of the new chemical entities and 42% of the anticancer drugs introduced into the market can be traced back to NPs [2]. In addition to plants and animals, microorganisms are a major resource for the discovery of new drugs. More than 50,000 microbial natural products (MNPs) have been obtained and have played an important role in drug discovery. The majority of these have been isolated from terrestrial-borne microbes [3]. However, after 50 years of intensive screening from terrestrial-borne microbes, the pace of MNPs’ discovery and development with a unique scaffold has dramatically declined over the last two decades. Meanwhile, the emergence of severe resistance to antibiotics in microbial pathogens, such as Gram-positive methicillin-resistant *Staphylococcus aureus* (MRSA) and vancomycin-resistant *S. aureus* (VRSA), and the current increase in the number of new diseases/pathogens, e.g., Gram-negative New Delhi metallo-beta-lactmase (NMD-1) bacteria have caused a resurgence of interest in the discovery of MNPs with unique scaffolds to meet the urgent demand for new drugs. Recent trends in drug discovery emphasize that marine microorganisms are a potentially productive source of novel secondary metabolites and have great potential to increase the number of marine NPs in clinical trials [4]. In contrast to the terrestrial environment, the oceans are a rich and relatively untapped reservoir of novel NPs. Over 15,000 structurally diverse NPs with an astounding assortment of bioactivities have been identified from marine environments since the 1970s [5]. This diversity has attracted researchers to screen MMNPs in drug discovery. Over 30 compounds derived from marine microbes such as didemnin B (Aplidine™) and thiocoraline are in clinical or preclinical studies for the treatment of different types of cancers [6]. However, the search for MMNPs has only just begun [6,7]. In this paper, we review the recent advances in MMNP discovery and development, especially addressing two important topics: (i) isolation and cultivation approaches of marine microorganisms, and (ii) strategies for the discovery and development of MMNPs.

## 2. Isolation and Cultivation of Marine Microorganisms

Marine microorganisms are a major source for MMNP discovery. Currently, 16 of 20 marine antitumor compounds under clinical trial are derived from microbial sources [8]. Therefore, isolation and cultivation of a new marine microorganism may be a shortcut to discover novel MMNPs. As indicated by microscopy and the fact that total cell counts are usually more than three orders of magnitude higher than the number of colony-forming units [9], the majority (>99.9%) of microorganisms from the environment do not form colonies on the nutrient-rich agar medium traditionally used for the isolation of marine microbes. This low culturability may reflect the artificial conditions inherent in most culture media, e.g., the lack of specific nutrient required for growth [10]. Despite the availability of varied molecular methods (e.g., PCR amplification of 16S rRNA) for analysis of microbial communities, cultivation-based analyses are far from redundant because a comprehensive characterization of physiological properties and a full assessment of application potential (e.g., bioactive compounds) may be undertaken only through the isolation of individual bacterial species in pure culture [11]. Hence, developing isolation and cultivation methods is a prerequisite for systematic investigation of marine microorganisms.

### 2.1. Isolation of Diverse Marine Microorganism Using Pretreatment Strategies

Pretreatment methods permit the isolation of specific groups of marine microorganisms, especially the less abundant bacteria. A variety of pretreatment methods including enrichment, physical, and chemical techniques (e.g., dry heat, exposure to 1%–1.5% phenol, sucrose-gradient centrifugation, and filtration through cellulose membrane filters) are employed to favor the isolation of specific genera and improve the recovery of these microorganisms [12,13,14,15,16]. These pretreatments eliminate or strongly reduce the risk of contamination, thereby facilitating the isolation of slow-growing marine microbes. To separate *Nocardia* from other actinomycete by centrifugation, Yamamura *et al.* [12] developed a sucrose centrifugation method for the relatively high specific isolation of *Nocardia*, a less abundant actinomycete. Most *Nocardia* cells were enriched in the 20% sucrose layer. By contrast, larger numbers of *Streptomyces* spp. were found in the 30%, 40%, and 50% sucrose layers. *Micromonospora* were observed in the 20% and 30% sucrose layers, but in relatively low numbers. The motile actinomycete *Actinoplanes* was only recovered from the 10% sucrose layer. Bredholdt *et al.* [13] used various pretreatment techniques (e.g., UV irradiation, super high frequency radiation, extremely high frequency radiation, and cold shock at −18 °C) to investigate the diversity of actinomycetes in the marine sediments of the Trondheim fjord, Norway. In addition to the predominant genera *Streptomyces* and *Micromonospora*, representatives of *Actinocorallia*, *Actinomadura*, *Knoellia*, *Glycomyces*, *Nocardia*, *Nocardiopsis*, *Nonomuraea*, *Pseudonocardia*, *Rhodococcus* and *Streptosporangium* genera were isolated, as well. Among them, the *Knoellia* and *Glycomyces* strains were the first to be isolated from a marine environment. Jensen *et al*. [14] utilized eight selective isolation techniques including dry/stamp, dry/scrape, dry/dilute, dilute/heat, dry/stamp + dilute/heat, and freeze/dilute to isolate actinomycete from 275 marine samples collected around the island of Guam. The dominant actinomycetes, including the seawater-dependent “*Salinospora*”, a new genus of the family Micromonosporaceae, were recovered. In addition, MAR2 and MAR3, members of two major new clades related to *Streptomyces* spp., were cultivated and appear to represent new genera within the Streptomycetaceae, which can be readily cultured using low nutrient media. Kjer *et al*. [16] gave a detailed description of the isolation and cultivation methods for fungi associated with various marine organisms (*i.e.*, sponges, algae, and mangroves). Marine-derived fungi can also produce a plethora of new bioactive secondary metabolites which are of interest as potential novel agents for medicinal use or in plant protection [16]. 

### 2.2. Selection and Design of Culture Media for Biodiversity of Marine Microbes

Energy sources, nutrients, and proper physicochemical conditions are necessary for microbial growth. Different microorganisms require different nutrients with the appropriate concentrations and forms. Many marine microbes have specific nutrient requirements for growth (e.g., sponge extract [17]) or chemical (e.g., siderophores [18], signal molecules, non-traditional electron donors, and electron acceptors [19]). Bruns *et al*. [20] employed artificial brackish water with different carbon substrates (agarose, starch, laminarin, xylan, chitin, and glucose) at low concentrations (200 μM each) as the growth medium to improve the cultivation efficiency of bacteria from the Gotland Deep in the central Baltic Sea. This method yielded significantly higher cultivation efficiencies (up to 11% in fluid media) in comparison to the results of previous studies [21]. Moreover, the addition of cyclic AMP (cAMP), *N*-butyryl homoserine lactone, or *N*-oxohexanoyl-DL-homoserine lactone at a low concentration of 10 μM can further significantly increase in cultivation successes. Among them, cAMP was the most effective inducer which led to cultivation efficiencies of up to 100% of total bacterial counts. 

**Figure 1:**
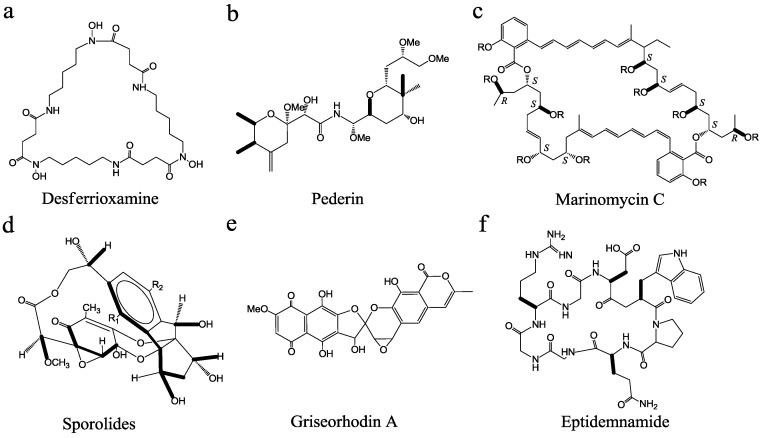
Structures of some marine microbial bioactive metabolites.

Other culture conditions, e.g., medium ionic strength, are important to marine microbial growth. Marine actinomycete genus *Salinispora* can produce bioactive secondary metabolites such as desferrioxamine (Figure 1a), saliniketals, arenamides, arenimycin and salinosporamide [22,23,24,25,26]. Tsueng *et al*. [27] observed that three species of *Salinispora*, *S. arenicola*, *S. tropica*, and *S. pacifica* require a high ionic strength for growth. Using both sodium chloride-based and lithium chloride-based media, *Salinispora* has a growth requirement for divalent ions magnesium and calcium, in addition to a growth requirement for ionic strength (8.29 to 15.2 mS/cm). Among them, *S. arenicola* has a lower growth requirement for ionic strength than *S. tropica* and *S. pacifica*. They also developed a potassium chloride-based salt formulation containing low sodium concentration (5.0 mM) to support the growth of *S. tropica* NPS21184 and its production of salinosporamide A (NPI-0052). Although *S. tropica* does not have a seawater growth requirement, it requires a specific combination of salts to provide a balance of salts and maintains a high enough ionic strength for growth [28]. 

### 2.3. Innovative Cultivation Approaches to Recover Less-Culturable or Unculturable Marine Microbes

Less than 1% of microorganisms can be cultured and these are not representative of the total phylogenetic diversity [29]; a significant focus for microbiologists today is thus to develop strategies to cultivate the uncultured majority of the microbial world for MMNPs’ discovery. Antitumor compounds pederin (Figure 1b), mycalamide A, and onnamide A discovered by an uncultivated bacterial symbiont of the marine sponge *Theonella swinhoei* [30] indicate the great potential of MMNPs’ discovery from such an uncultured majority. Different studies in recent years demonstrated that some “not-yet-cultured” species can be grown by the refinement of classical approaches. The nutrient-rich culture media may favor the growth of faster-growing microbes at the expense of slow-growing species which always thrive in nutrient-poor environments and may be inhibited by substrate-rich conventional media [11]. Consequently, culture mimicking the natural environment is critical to recover the uncultivated microorganisms. For instance, seawater has been used to culture previously uncultivated microbes, e.g., the ubiquitous SAR11 marine bacterioplankton clade [31,32]. Moreover, dilute nutrient media strategies are successfully applied in the cultivation of previously unculturable microbes from various marine habitats [10,32,33]. Stephanie *et al.* [33] isolated and cultivated four unique cell lineages belonging to previously uncultured or undescribed marine *Proteobacteria* clades at *in situ* substrate concentration (three orders of magnitude less than common laboratory media). These four unique cell lineages were related to the clades SAR11 (α subclass), OM43 (β subclass), SAR92 (γ subclass), and OM60/OM241 (γ subclass). In addition, certain microbes under laboratory conditions can be successfully cultivated only in the presence of other microorganisms (denoted as helper strains). Chemicals released from helper microbes are often growth-stimulating factors for the unculturable strains. Thus, the culture supernatants or cell-free extracts from helper strains were often used as the growth stimulators to the unculturable species [34,35,36,37].

Many microorganisms are very slow-growing in the marine environment; extended incubation time is thus prerequisite for the cultivation of such microbes at low substrate concentrations in defined media, with the added benefit of reducing the bacterial competition by the faster-growing members within mixed populations [11]. Song *et al*. [32] applied a modified dilution-to-extinction cultivation with prolonged incubation at low temperature to isolate the major bacterioplankton lineages in the East Sea, Western Pacific Ocean. Extinction cultures belonging to the SAR11, *Roseobacter*, OM43, and SAR92 clades were isolated from the plate incubation for 20 and 24 weeks. Other conditions, such as growth factors and signaling molecules, are also important for growing the “not-yet-cultured” species [11,29]. Recently, three reviews [11,29,38] have explicitly discussed the possible reasons for unculturability and various strategies for culture of unculturable microorganisms. Although many “not-yet-cultured” species can be grown and the molecular mechanisms of unculturability have been unraveled, developing new cultivation strategies is still an important task for all microbiologists and plays a key role in identifying new species.

Various techniques, such as high throughput screening (HTS), diffusion chamber system [37,39], encapsulation method [34,40], soil substrate membrane system [41,42,43,44], filtration method [45], density-gradient centrifugation, extinction dilution [46], and fluorescence-activated cell sorting (FACS) [46] will also have a significant effect in recovering as-yet-uncultivated species in recent years. Zengler *et al*. [10] combined cells’ encapsulation in gel microdroplets for massively parallel microbial cultivation under low nutrient flux conditions and detected microdroplets containing microcolonies by flow cytometry. Kaeberlein *et al*. [39] designed a diffusion chamber and allowed the substances from the natural environment across a membrane which successfully isolated the previously uncultivated microorganisms from marine sediment. They observed that these isolates formed colonies on artificial media only in the presence of other microbes. Similar diffusion chambers were also applied in rarely cultivated bacteria from marine [37]. One of the recently developed, innovative techniques is the substrate membrane used for the microcolony cultivation of uncultivated bacteria system [42]. This system includes a polycarbonate membrane support and soil extract as a substrate. It allows the microcultivation of novel bacterial strains, enables the detection of live microcolonies using viability staining, and micro-manipulates the isolation of these colonies [41].

Taken together, finding the structurally unique compounds with interesting biological activities will still require new approaches and techniques of isolation and cultivation to recover more cultivated and as-yet-uncultivated microbial species. 

## 3. Discovery and Development of MMNPs

The discovery and development of novel MMNPs is encouraged not only by the isolation of new marine microbes but also by the novelty of screening strategies. In this section, five screening strategies, including (i) conventional screening, (ii) metagenomics, (iii) genomics, (iv) combinatorial biosynthesis, and (v) synthetic biology are introduced for MMNPs’ discovery and development. An overview of the key studies of MMNPs discovered via different methods is provided in Table 1. 

**Table 1:** Representative examples of MMNPs discovered byvarious methods.

Compounds	Host	Method	Activity	Reference
marinomycin	*Marinispora* sp. CNQ-140	bioactivity-guided screening	antitumor	[47]
medermycin	*Streptomyces* sp. 16	gene-guided screening	antimicrobial and antitumor	[48]
pederin	uncultured *Pseudomonas* sp.	metagenomics	antitumor	[30]
salinilactam A	*Salinospora tropica*	genomics	antitumor	[25]
salinosporamide X1/X2	*Salinospora tropica*	combinatorial biosynthesis	proteasome inhibitor	[49]
eptidemnamide	*Prochloron* spp.	synthetic biology	antitumor	[50]

### 3.1. MMNPs Discovery via Conventional Screenings

The conventional screening methods include: (i) bioactivity-guided screening and (ii) gene-guided screening. The bioactivity-guided screening can directly detect the activity (e.g., antimicrobial, antitumor, antiviral, and antiparasitic activities) using the culture supernatant or extract of cell pellet. For instance, antitumor antibiotics with a new structural class, marinomycins A–D (Figure 1c), were isolated from the saline culture of *Marinispora* sp. CNQ-140 based on significant antibacterial activities (0.1–0.6 μM of MIC values) against drug-resistant pathogens (e.g., MRSA) and impressive and selective cancer cell cytotoxicities (0.2–2.7 μM of MIC_50_ values) against six melanoma cell lines in the National Cancer Institute’s NCI-60 cell line panel [47].

To identify new sources of bioactive secondary metabolites, gene-guided screening has been deployed to search target genes associated with NPs biosynthetic pathways, e.g., the fragments between ketosynthase and methylmalonyl-CoA transferase of polyketides (PKS) type I [51], enediyne PKS ketosynthase gene [52], *O*-methyltransferase gene [53], P450 monooxygenase gene [54], polyether epoxidase gene [55], 3-hydroxyl-3-methylglutaryl coenzyme A reductase gene [56], dTDP-glucose-4,6-dehydratase (dTGD) gene [48], and halogenase gene [57]. When combined with homology-based searches and phylogenetic analyses, gene-based screening offers the greatest potential to predict the production of interesting, new secondary metabolites harbored by isolates or environments. This predictive capability provides a simple and rapid method to avoid the isolation of known compounds or to identify strains that produce compounds within a desired structural class [58]. Chen *et al.* [48] investigated the distribution of dTGD gene and diversity of the potential 6-deoxyhexose (6DOH) glycosylated compounds from 91 marine sediment-derived bacteria, representing 48 OTUs and belonging to 25 genera, by PCR. Eighty-four percent of the strains were dTGD gene positive, suggesting that 6DOH biosynthetic pathway is widespread in these marine sediment-derived bacteria. BLASTp results also indicated a high chemical diversity of the potential 6DOH glycosylated compounds. The results demonstrated that phylogenetic analysis of dTGD gene is useful for structure prediction of glycosylated compounds from newly isolated strains and can therefore guide the chemical purification and structure identification process such as medermycin and chromomycin A3 [48]. Hence, gene-guided screening provides a bioinformatic assessment of the secondary metabolite biosynthetic potential in the absence of fully assembled pathways or genome sequences. This simple and rapid prediction, which possesses new secondary metabolites or known compounds in the strains, can improve the process of MMNP discovery by providing a method to prioritize strains for fermentation studies and chemical analysis.

**Figure 2:**
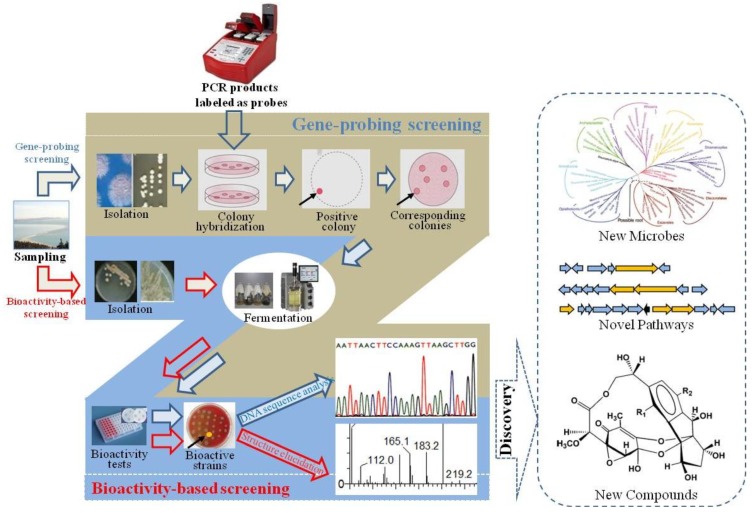
The combined strategy of gene-based screening and bioactivity-based screening for marine microbial natural products’ (MMNPs) discovery.

However, owing to the disadvantages of bioactivity/gene-guided screening, the combined strategy of gene and bioactivity screening (Figure 2) could be more powerful to obtain valuable strains with the potential to synthesize novel bioactive compounds. Zhang *et al.* [59] investigated nonribosomal peptide synthetase (NRPS) genes by PCR for 109 bacteria isolated from four South China Sea sponges. Fifteen bacteria were found to contain NRPS genes and grouped into two phyla Firmicutes (13 of 15) and Proteobacteria (2 of 15) based on 16S rDNA sequences. Most of the NRPS fragments (11 of 15) showed <70% similarity to their closest relatives based on the phylogenetic analysis of the conserved A domain, thus suggestive of the novelty and high diversity of these NRPS genes. All of the 15 bacteria with NRPS genes had antimicrobial activities, with most of them exhibiting broad-spectrum activities against fungi and bacteria, indicating the chemical diversity of biologically active metabolites of sponge-associated bacteria and the possible role of bacterial symbionts in the host’s antimicrobial chemical defense [59]. In brief, the combined gene and bioactivity strategy will be useful to obtain novel MMNPs.

### 3.2. MMNPs Discovery via Metagenomics to Bypass the Culture-Dependent Bottleneck

More than 99% of the microorganisms are not readily cultured in the laboratory. Culture-independent methods are thus required in order to discover the majority of microorganisms [60]. Metagenomics enables direct access to the genomes of whole environmental microorganisms by total environmental DNA (eDNA) extraction. It is an effective way to access NPs encoded by the genomes of previously uncultured microbes through introduction of eDNA into a suitable host and screening of these large eDNA libraries for bioactive clones [61]. eDNA libraries extracted from microbial populations in extremely complicated or unexplored circumstances are thus invaluable sources for MMNP discovery [62,63]. Discovery of unique structures with various bioactivities such as terragines, violacein, indirubin and turbomycins through the metagenomic method has proven that this technology is a good alternative for exploiting uncultivable microbes for NP drug discovery [5]. 

Currently, function driven analysis (e.g., bioactivity assay) and sequence-driven analysis (e.g., DNA probe) are two main approaches for eDNA library screening [61,62,64]. Sequence-based screening using homologous PCR or clone hybridization identifies the indispensible genetic elements for the intact cluster assembly [60]. Based on sequence-driven analysis, Feng *et al.* [65] used transformation-associated recombination to reassemble two characterized eDNA clones (named AB649 and AB185) into a complete Type II PKS biosynthetic pathway and heterologously expressed in *S. albus*. Four novel metabolite fluostatins, C, F, G and H, were detected in assembled clones, while none were found in AB649 cultures alone [65]. 

In addition, the development of diverse model microbial systems that can serve as heterologous hosts for eDNA expression could expand the repertoire of NPs. Craig *et al*. [66] explored β-Proteobacterium *Ralstonia metallidurans* as a new model system for the expression of eDNA library. Interestingly, cosmids conferring the production of novel pigmented/antibacterial metabolites in *R. metallidurans* clones did not function in *E. coli* [66]. Novel compounds such as indigo, patellamide, and pederin [30] with a simple biosynthetic diagram were isolated by this new system. Thus, diverse model hosts will expand the collection of metabolites found in future metagenomic discovery.

The drawbacks of metagenomics may be the inability of efficient acquisition of intact gene fragment and incompatibility of expression elements such as promoter in a heterologous host. Although it is still too early to make any conclusion about metagenomics-based MMNP discovery, we believe that rapid advances in synthetic biology, e.g., large DNA fragment assembly techniques for artificial genome synthesis and synthetic microbial chassis suitable for different classes of MMNP biosynthesis, will greatly facilitate the active expression of the entire eDNA cluster.

### 3.3. Mining Diverse Biosynthetic Clusters of MMNPs via Genomics Strategies

Genomics has been applied to MNP discovery because secondary metabolites such as PKS, NRPS, and PKS–NRPS hybrids are often biosynthesized by large multifunctional synthases which sequentially assemble small carboxylic acid and amino acid building blocks into their products [25]. Advances in DNA sequencing and bioinformatic technologies make it possible to rapidly identify the gene cluster of bioactive compounds and *in silico* predict their chemical structure based on genomics information. These structural predictions can be used to guide compound purification, structure confirmation, and identify new chemical entities [67]. 

To date, more than 450 MNP gene clusters have been successfully identified by genome sequence tags (GSTs) probe through the genome scanning approach [68]. Zazopoulos *et al*. [68] used this method to isolate an enediyne (a potent class of antitumor antibiotics) gene cluster from a variety of marine actinomycete strains to produce enediynes. Comparative analysis of five biosynthetic loci representative of enediynes revealed a conserved cassette of five gene clusters, including a novel family of PKS. The enediyne PKS is involved in the formation of the highly reactive chromophore ring (or “warhead”) structure in all enediynes. Genome scanning analysis indicated that the enediyne warhead cassette is widespread among actinomycete. 

The number of gene clusters responsible for NP biosynthesis is dramatically higher than the number of known metabolites in microorganisms by genome sequencing [69]. For instance, genome analysis of actinomycete reveals numerous cryptic gene clusters [70,71,72]. Each actinomycete strain appears to have the potential to produce approximately 20 NPs on average, but the traditional screening routinely identifies only approximately two NPs per strain [69]. In the case of *S. tropica*, the compounds produced by this marine bacterium include the potent proteasome inhibitor salinosporamide A [73], the unprecedented halogenated macrolides sporolides A and B (Figure 1d) [74], lymphostin and salinilactam. Udwary *et al*. [25] identified all secondary metabolic biosynthetic gene clusters of *S. tropica* CNB-440 by genome sequencing. The majority of the 17 biosynthetic clusters are novel. This strain is found to possess the most diverse polyketide biosynthetic mechanisms, as well as the largest percentage (~9.9%) of a genome devoted to NPs biosynthesis. Bioinformatic analysis has not only been used to facilitate the structure elucidation of the polyene macrolactam salinilactam A, but its structural analysis has aided the genome assembly of the highly repetitive *slm* loci [25]. Winter *et al*. [75] have summarized the recent discoveries in this area and discussed the potential future of the field. Currently, more than 50 actinomycete genomes are being sequenced worldwide [76,77], which will make genomics increasingly attractive. Hence, with the development of various genome mining approaches, genomics will be more powerful in the discovery of MMNPs.

### 3.4. Diversified MMNPs via Combinatorial Biosynthesis

The complex structures of NPs represent a challenge for the generation of derivatives by chemical synthesis. To overcome this problem, combinatorial biosynthesis is a useful tool to increase the chemical diversity of NPs. It involves the genetic manipulation of NPs’ biosynthetic cluster to obtain new/altered structures that would be difficult to synthesize using other methods. It is therefore an efficient complement to traditional microbial drug development programs. Compared with the derived elements of terrestrial sources, MMNPs’ biosynthetic elements have their own unique aspects, such as halogenase and other novel enzymes. Unnatural products can be yielded by heterologous expression of biosynthetic genes from diverse origins through combinatorial biosynthesis [78]. In the salinosporamide A biosynthesis, the chlorinase SalL halogenates *S*-adenosyl-l-methionine (SAM) to generate 5′-chloro-5′-deoxyadenosine (5′-ClDA) in a rarely occurring nucleophilic substitution analogous to that of the fluorinase in the fluoroacetate producer *S. cattleya* [79]. The substrates of SalL are also bromide and iodide, not fluoride. Eustaquio *et al.* [79] added synthetic 5′-fluoro-5′-deoxyadenosine (5′-FDA) to a salL-knockout mutant of *S. tropica* devoid of salinosporamide A and led to the synthesis of fluorosalinosporamide, a new salinosporamide derivative. Moreover, a new shunt in the phenylalanine pathway to L-3-cyclohex-2′-enylalanine (CHA) residue in salinosporamide A (SalX disruption in *S. tropica* mutant) through combinatorial biosynthesis enabled the generation of not only antiprotealide, but also other unnatural salinosporamide derivatives (salinosporamide X1 and salinosporamide X2) with C5 modifications in the salinosporamide family of potent proteasome inhibitors [49]. 

Despite the many successes of combinatorial biosynthesis [50,80], there is an obstacle because the productivity of the engineered compound is often lower than that of the parent MMNPs. In addition, although the range of possible structural modifications is extensive, compound libraries with huge diversity are still not available through combinatorial biosynthesis [69]. Thus, these problems that exist in combinatorial biosynthesis will likely be overcome within the next decade. 

### 3.5. Diversified MMNPs via Synthetic Biology

Although the great structural diversity in MMNPs’ libraries provides a drug pool for public health, MMNPs commonly accumulate at very low levels in the native producers. With the advance of microbial cultivation and fermentation technologies, more MMNPs can be prepared to support their structure elucidation, activity analysis, and even clinical trials. However, due to the higher production cost of MMNPs, ordinary people still cannot afford the therapeutic expense of MMNPs. In addition, more and more speculated MMNPs discovered by genome or environmental DNA sequence mining are also waiting to be unraveled. With the rapid development of synthetic biology, it can, therefore, be a promising strategy to improve the production of known compounds or activate the silent gene clusters. Based on the development of genome-wide genetic manipulation techniques such as hierarchical conjugative assembly genome engineering (CAGE) [81] and multiplex automated genome engineering (MAGE) [82], and the functional definition of abundant genetic materials (e.g., functional genes derived from different sources, controllable regulatory elements [83,84], and synthetic RNA/protein scaffolds [85,86]), synthetic biology can assemble natural or artificial biosynthetic pathways in the host microbes for MMNPs. The high yield of erythromycin precursor 6-deoxy-erythronolide B, taxol precursor taxadiene and artemisinin precursor amorphadiene in the surrogate hosts [87,88,89,90,91,92,93] are the best examples given the successes of synthetic biology, suggesting its great potential for the production of valuable MMNPs.

As a concept of synthetic biology, in addition to efficient DNA manipulation and transfer technologies, compatibilities between host microbes and synthetic materials of the desired product including pathway genes expression, enzymes activity, and precursors supply are important considerations for host selection. Although NPs’ biosynthesis has high similarity between terrestrial and marine microbes, the development of marine-derived hosts (e.g., marine derived actinomycete, cyanobacteria, and symbiotic fungi) will be valuable in the attempt to achieve heterologous expression of MMNPs. Genome-minimized microorganisms [94], whose non-essential DNA regions have been deleted, are also promising biological chassis for MMNPs. All developments of the above researches and techniques pave the road for MMNP production using heterologous expression. 

Heterologous expression of complete NP gene clusters is an elegant strategy to elucidate the role of genes and gene clusters involved in metabolite biosynthesis [95]. Piel *et al.* [78] isolated *Streptomyces* sp. JP95 from the marine ascidian *Aplidium lenticulum* at Heron Island, Queensland, Australia. The telomerase inhibitor griseorhodin A (Figure 1e) produced by *Streptomyces* sp. JP95 is probably the most heavily oxidized bacterial polyketide known and features a unique epoxyspiroketal moiety crucial for its activity [78]. The griseorhodin biosynthetic cluster encodes an unprecedented number of functionally diverse oxidoreductases (encoded by 11 various ORFs), which are involved in the oxidative modification of a polyaromatic tridecaketide precursor by cleavage of three carbon–carbon bonds. Unexpectedly, *Streptomyces* sp. JP95 is highly refractory against the introduction of foreign DNA by a variety of methods, thus precluding knockout studies to confirm that the *grh* cluster is involved in griseorhodin biosynthesis. An alternative strategy is heterologous expression of the complete *grh* cluster in *S. lividans* on a suitable shuttle cosmid. As a consequence, the engineered strain *S. lividans* ZX1 (pMP31a) can successfully produce griseorhodin A and three other related compounds. Another successful case is that a new cyclic peptide eptidemnamide (Figure 1f) was produced by an engineered *E. coli* through replacing the ulithiacyclamide (5)-coding region from *patE2* (responsible for heterocyclization of cysteine, serine and threonine residues, and *N*-terminal to *C*-terminal cyclization to afford the final patellamides) with a wholly artificial construct and expression of the *pat* cluster (responsible for patellamide biosynthesis) from the obligate cyanobacterial symbionts *Prochloron* spp., thereby demonstrating for the first time that the whole biosynthetic cluster of MMNPs can be functionally expressed in the surrogate host [50]. Despite the fact that synthetic biology is a new discipline and its theory and methods should be further developed, there is no doubt that synthetic biology will trigger new era of MMNP development [96].

## 4. Conclusions

Although some novel compounds from marine microorganisms are under investigation in preclinical/clinical trials, there is still an urgent unmet medical need for the development of novel NPs, and MMNPs appear as the most promising and endless source for drug development. In addition to the aforementioned methods, other innovative approaches, such as ribosome engineering [97,98] and the OSMAC (one strain, many compounds) method [99,100], will also strongly support MMNP development. We believe MMNPs can be better understood and discovered in the next decade by a combination of both conventional and innovative approaches.
